# Detoxifying Antitumoral Drugs via Nanoconjugation: The Case of Gold Nanoparticles and Cisplatin

**DOI:** 10.1371/journal.pone.0047562

**Published:** 2012-10-17

**Authors:** Joan Comenge, Carmen Sotelo, Francisco Romero, Oscar Gallego, Agustí Barnadas, Tomás García-Caballero Parada, Fernando Domínguez, Víctor F. Puntes

**Affiliations:** 1 Catalan Institute of Nanotechnology (ICN), Universitat Autònoma de Barcelona (UAB), Bellaterra, Barcelona, Spain; 2 International Iberian Nanotechnology Laboratory (INL), Braga, Portugal; 3 Department of Physiology, Faculty of Medicine, Santiago de Compostela University, Santiago de Compostela, Spain; 4 Molecular Science Institute, University of Valencia, Paterna, Spain; 5 Oncology Department, Sant Pau Hospital, Barcelona, Spain; 6 Department of Morphological Sciences, School of Medicine-University, Clinical Hospital, Santiago de Compostela University, Santiago de Compostela, Spain; 7 Institució Catalana de Recerca i Estudis Avançats (ICREA), Barcelona, Spain; Argonne National Laboratory, United States of America

## Abstract

Nanoparticles (NPs) have emerged as a potential tool to improve cancer treatment. Among the proposed uses in imaging and therapy, their use as a drug delivery scaffold has been extensively highlighted. However, there are still some controversial points which need a deeper understanding before clinical application can occur. Here the use of gold nanoparticles (AuNPs) to detoxify the antitumoral agent cisplatin, linked to a nanoparticle via a pH-sensitive coordination bond for endosomal release, is presented. The NP conjugate design has important effects on pharmacokinetics, conjugate evolution and biodistribution and results in an absence of observed toxicity. Besides, AuNPs present unique opportunities as drug delivery scaffolds due to their size and surface tunability. Here we show that cisplatin-induced toxicity is clearly reduced without affecting the therapeutic benefits in mice models. The NPs not only act as carriers, but also protect the drug from deactivation by plasma proteins until conjugates are internalized in cells and cisplatin is released. Additionally, the possibility to track the drug (Pt) and vehicle (Au) separately as a function of organ and time enables a better understanding of how nanocarriers are processed by the organism.

## Introduction

### 1. NanoOncology

The global death rate from cancer has declined only marginally over the past several decades, in contrast to dramatic reversals in death rates from heart disease, stroke, and infectious disease over the same time period [1]. In this context, nanotechnology emerges as a “disruptive technology” with a great potential to contribute to improve cancer treatment by generating new diagnostic and therapeutic products [2]–[5]. Thus, nanotechnology has been proposed to enable researchers to combine a series of advances; creating nanosized particles that may contain drugs designed to kill tumors together with targeting compounds designed to home in on malignancies [6]–[8], and imaging agents designed to light up even the earliest stage of cancer or monitor its treatment [9]. Thus, functionalized nanoparticles could deliver multiple therapeutic agents to tumor sites in order to simultaneously attack multiple points in the pathways involved in cancer. However, despite the plethora of nanoparticles (NP), organic or inorganic, and conjugated chemotherapeutic agents which have shown promising results *in vitro*, the precise behavior of these conjugates *in vivo* is still rather unknown, with controversy about disparities between the *in vitro* and *in vivo* results or results from different laboratories. There are indications that small modifications of the nature of the conjugate have a strong influence on conjugate interactions [10], protein corona formation [11], [12], aggregation [13], degradation [14], and consequently biological behavior during the full life cycle of the conjugate inside the body [15]. By attaching the drug to the NP, its physicochemical fate is modified. Thus, nanocarriers can strongly contribute to modifications in pharmacokinetics (Table 1) and biodistribution, by leading the drug through different pathways depending on the physicochemical properties of the nanocarrier (e.g., size and surface charge), which is especially appealing in the case of very toxic drugs [16], [17]. Inside the body, pores smaller than 1 nm have been reported in the tight junctions on certain continuous capillaries (including the central nervous system, i.e., blood-brain barrier, placenta and testis barrier) while continuous capillaries (muscle, lung, skin) have pores of 6 nm [18]. Fenestrated capillaries (kidney, intestine, some endocrine and exocrine glands) have pores up to 50–60 nm, usually closed by a diaphragm [18]. Finally, discontinuous capillaries (liver, spleen, bone marrow) have pores between 100–1000 nm, which allow the passage of macromolecules between plasma and interstitium [18]. Thus, small molecules (below 6 nm, the majority of drugs) leak in and out from the blood vessels and are rapidly (in minutes) cleared from blood via the kidneys [19] while the passive transport of macromolecules through these porous is negligible. Thus a NP sized between 6–40 nm may follow protein paths to finally accumulate in organs of the mononuclear phagocyte system, especially the liver and spleen, as do proteins and protein aggregates [20], while larger sizes of NP are easily recognized by the immune system and also end up in liver and spleen but within a shorter time [21]. It is worth noting here that blood vessel permeability changes in diseases such as inflammation and cancer [22]. In cancer, the rapid growth of tumor results in leaky vessels. These fenestrated vessels allow macromolecules and NPs to permeate through the tumor. In addition, the nanoparticles are retained due to the lack of a functional lymphatic system. This effect (Enhanced Permeability and Retention effect, EPR) is widely reported in the literature [23], [24] and has been exploited to passively accumulate nanocarriers in tumors [4]. Other described pathways are more complex and include the use of migrating macrophages as transporters of NPs and drugs [25]. In all cases, surface modifications allow the modification of this size-dependent fate, for example, by making small NPs recognizable by the immune system [26] or shielding the large ones by means of chemical modification such as pegylation [27].

**Table 1:** Key words in pharmacology.

**Efficacy**	The ability of a drug to produce the desired therapeutic effect.
**Efficiency**	The ability of a drug to produce few or no side effects while still performing its work.
**Pharmacokinetics**	How the body affects a specific drug after administration. *What the body does to the drug*.
**Pharmacodynamics**	The study of the biochemical and physiological effects of drugs on the body. *What the drug does to the body*.

Beyond oncology, small inorganic NPs the size of a small protein (5–30 nm) are making their way towards the clinic: AuNPs are used in cell imaging [28], targeted drug delivery [29], [30], as photothermal agents for hyperthermia [31], and in other proposed diagnoses and therapies [32]. AgNPs display a biocidal effect [33] that is currently applied in commercial products such as hospital equipment and devices. Magnetic NPs are present in various biomedical applications, *e.g.,* the early detection of cancer, diabetes, and atherosclerosis [34]. CeO_2_NPs are being used in biomedicine as an antioxidant to treat disorders caused by oxygen radicals, such as retinal degeneration [35] or cardiomyopathy [36]. Non-inorganic nanomaterials have also reached the clinics, e.g., Doxil, which is a liposomal formulation (hundreds of nanometers in size, biocompatible and biodegradable) of doxorubicin that increases the solubility of the active ingredient and modifies the dosing by sustaining it over time.

### 2. The Case of Cisplatin

**Figure 1:**
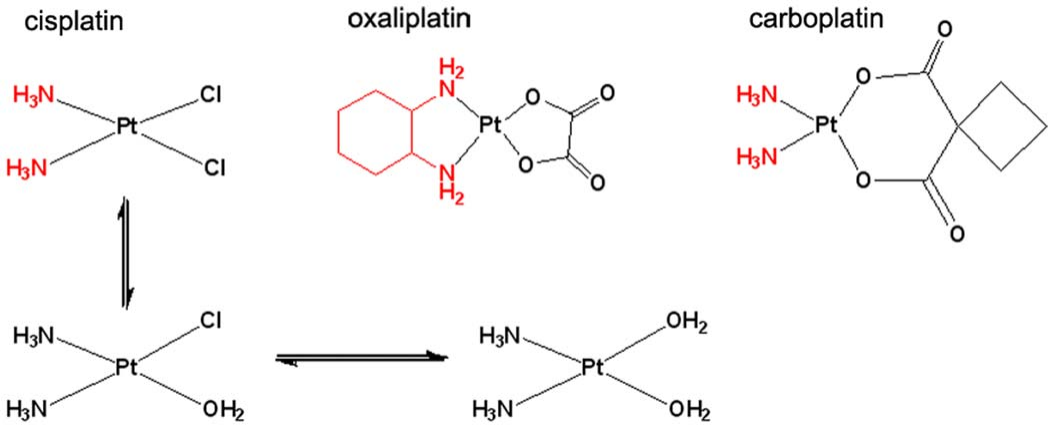
Different platinum anticancer drugs approved by the FDA. The active part of each drug is drawn in black; in all cases it is characterized by the presence of good leaving groups that will allow the Pt atom to bind the target. In red, the amines play a role in the modulation of the activity and distribution of the drug. In the case of cisplatin, the equilibrium that spontaneously occurs inside the cell (where the chloride concentration drops from 100 mM to 4 mM) is also shown.

Platinum compounds (Figure 1) are a paradigm in anticancer drugs. Cisplatin or cis-diamminedichloroplatinum(II), [PtCl_2_(NH_3_)_2_], was originally synthesized in 1845, but not until 1970 was its antitumor activity established [37]. Today cisplatin is used to treat various types of cancers (i.e., non-small-cell lung cancer, ovarian cancer, germ cell tumors, osteosarcomas, etc.), with a cure rate as high as 90% in testicular cancer [38]. The platinum complex reacts *in vivo* to form adducts with DNA, which ultimately trigger apoptosis [39]. It has been proven that, after both passive and active cellular uptake, cisplatin may react with the N^7^ atom of purine bases in DNA [38]. However, chronic cisplatin usage results in resistance via several possible mechanisms including increased interactions with metallothioneins and glutathione, which deactivate the drug, as well as increased DNA repair and/or cisplatin efflux [40]. To counteract resistance, which lowers the efficiency of cisplatin significantly, very high systemic doses of cisplatin should be administered. Unfortunately, such high doses of cisplatin result in severe systemic toxicity and poor patient compliance, including nausea/vomiting, renal toxicity, gastrointestinal toxicity, peripheral neuropathy, asthenia, and ototoxicity, which thus limit its clinical use [40], [41]. Of all the toxicities induced by cisplatin, nephrotoxicity is considered to be the dose-limiting factor [39]. Such side effects make it impossible to achieve the full benefit of the treatment in a large number of patients [42]. In humans, cisplatin treatment generally involves series of intravenous injections administered every 3–4 weeks at a dose of 50–120 mg/m^2^ (1.2–2.7 mg kg^−1^). In addition to the undesired side effects, there is also a loss of drug activity in the body associated with poor circulation and poor delivery to the tumor, as well as deactivation mechanisms that irreversibly alter the chemistry of these molecules before reaching the tumor cells [41]. Since its discovery, many attempts to find derivatives of cisplatin have looked for both reduced side effects and modified body distribution (in order to target different organs), rather than improving cisplatin efficacy [40], [43]. Here, second-generation platinum drugs such as carboplatin and oxaliplatin represent an improvement in some cancer treatments, for lung and colorectal respectively, although the limitations observed for cisplatin have not been entirely overcome [39], [40].

### 3. Carrying Cisplatin

Recent efforts have been focused on targeting the tumor by using drug delivery systems to avoid the organs to which cisplatin is toxic. As the kidney is responsible for filtration and removal from the blood of molecules smaller than 50 KDa, which corresponds to molecular diameters of around 6 nm, any larger delivery vehicle will divert the drug away from the kidney [19]. Additionally, NPs accumulate in the tumor due to the EPR effect [23], [24]; which is known to be strongly size-dependent [44], [45]. Therefore, when the target is a solid tumor, nanometer-sized carriers are expected to be passively accumulated on it. This case also applies when cisplatin is bound to albumin. Up to 90% of the administrated cisplatin is known to bind irreversibly to albumin [46] and then reach the tumor by EPR; however, this form of cisplatin is inactive and has no biological effect [47]. Thus, a properly designed nanocarrier will not only transport the cisplatin to the tumor, but also protect it against plasma deactivation.

In this context, approaches based on the encapsulation and transport of cisplatin have emerged. Sterically stabilized polymeric nanoparticles, which have excellent stability in plasma, a much longer circulation time, better efficacy, and lower toxicity than free cisplatin have been reported [6], [7], [48], [49]. Such vehicles include lipid capsules [50] or polymers as in Prolindac®, which has a 22 kDa hydroxypropylmethacrilamide copolymer as a backbone and then a pH sensitive glycine chelator linker [51]. Other examples include soluble CNTs [52], carbon nanohorns [53], and Fe_3_O_4_ NPs [54]. Similarly, CytImmune Corp. is developing AuNPs as a carrier for TNF-α and doxorubicin.AuNPs have been recently proposed as scaffolds for cisplatin due to their controlled and reproducible synthesis and conjugation to cisplatin as well as the high loading of drug achieved [55]. Not only cisplatin, but other Pt derivatives, such as Pt (IV) prodrugs, have also been loaded on AuNPs with maintenance of the anticancer effect [56]. In closely related work, Ren *et al.* [57]–[59] reported the adsorption of commercial cisplatin to gold colloids via ionic interactions. In the case of adsorption via ionic interactions on the surface of the nanomaterials, an uncontrolled rapid liberation of the drug is observed as soon as the conjugates are dispersed in highly ionic media such as serum [60]. In many of the reported systems, colloidal stability in the working environment was an issue.

## Materials and Methods

### Ethics Statement

Mice were obtained from the Central Animal House of the Santiago de Compostela University (USC), a registered animal facility that maintains the animals under welfare and ethical conditions complying with the European (86/609/CEE) and Spanish (RD223/88 and OM 13/10/89) laws on laboratory animal care and handling. In order to ameliorate suffering, mice were anaesthetized with 2,2,2-tribromoethanol-2-methyl-2-butanol (Avertine®, Sigma Aldrich) before xenotransplantation and before receiving the different treatments reported here. After the treatments, mice were euthanized by CO_2_ inhalation. The biological work related to the results reported in this article had the approval of the Ethical Committee of the USC and followed the European and the Spanish legislations. 5 mL of blood were obtained with informed written consent from a normal control (JC, coauthor of the present work) and with approval from the Ethical Committee of the USC.

### Synthesis and Conjugation of AuNP-cisplatin

13 nm AuNPs were synthesized following a seeded-growth approach and loaded with cisplatin in a two-step conjugation as described in the literature [61] and in the Supplementary Information (Text S1). There are several reasons for using this synthesis: Control of the size is better than that achieved by classical methods, the size distribution is very narrow and the concentration of AuNPs is higher. Moreover, and according to our experience, the reproducibility is far beyond that achievable by using classic protocols. For more details, one can read our recent work [61].

### In vitro Stability and pH-dependent Release

17 µL of AuNP-cisplatin (2.75×10^14^ NP ml ^–1^) were added to 500 µL of human blood and gently mixed over 24 h. Colloidal stability was assayed by using DLS and UV-Vis spectroscopy. The UV-Vis spectra of all the AuNPs in the present work were recorded from 300–800 nm at 0.5 nm intervals. When needed, appropriate dilutions were performed to overcome the saturation limit of absorbance. The release of cisplatin in physiological conditions was performed in solutions consisting of 50 mM buffer species, 120 mM NaCl, and 20% Foetal Bovine Serum (FBS). Buffer species were HEPES for pH 7.6, MES for pH 6.2 and 4.4, Glycine/HCl for pH 4.4 and 3.8 and Acetate for pH 4.4. 100 µL of AuNP-cisplatin were added to 900 µL of the corresponding buffered solution and mixed over differing lengths of time (2, 8, 24, and 144 hours). AuNPs were removed by means of two centrifugation steps (15 minutes, 35000 rcf). The amount of Pt in the supernatant was analyzed by using ICP-MS.

### Pt Cell Internalization and DNA Accumulation

The human lung carcinoma derived cell line A549 was obtained from American Tissue Culture Collection (ATCC) and cultured in a 1∶1 mixture of Dulbeccós Modified Eaglés Medium (DMEM, Sigma-Aldrich) and Hams F-12 Medium (Sigma-Aldrich), supplemented of 10% (v/v) foetal bovine serum (FBS, GIBCO-Invitrogen) and 1% (v/v) of L-glutamine, penicillin and streptomycin solution (GPS, Sigma-Aldrich).To quantify Pt cell internalization, 5×10^5^ cells were plated in 60-mm-diameter plates (Falcon) and 24 hours later, medium was changed for treatments diluted in culture medium: free cisplatin or cisplatin conjugated to AuNPs (1.67 µg cisplatin mL^−1^ in both cases). After 0.5, 1, 3, or 24 hours of treatment, cells were trypsinized and centrifuged, supernatant was removed and cells were resuspended in 0.5 mL 65% HNO_3_ (Merck). The amount of Pt was determined by using ICP-MS (Bruker 820-MS). To quantify cisplatin bound to DNA, 10^6^ cells were treated as above and DNA extraction was performed by using a commercial kit (QIAamp DNA Blood Mini Kit, QIAGEN). DNA was finally resuspended in 200 µL of water. This solution was used to determine both the amount of DNA and Pt by using UV-Vis spectroscopy and ICP-MS, respectively.

### TEM Cell Imaging

A549 cells were centrifuged for 15 min at 3000 rpm, fixed by immersion for 45 min in 2.5% glutaraldehyde in sodium cacodylate buffer (0.15 M, pH = 7.3), postfixed with 2% osmium tetroxide in the same buffer, dehydrated, embedded in LRWhite resin, Medium Grade, and sectioned. Sections were stained with uranyl acetate and lead citrate and examined in a Zeiss 902 electron microscope (Carl Zeiss, F.R.G.) at 80 kV accelerating voltage.

### Biodistribution in Mice

*In vivo* biodistribution was measured by treating two groups of 12 female tumor-bearing SCID mice (three mice per point). All mice received a single intraperitoneal (ip) dose of 4 mg cisplatin kg^–1^ mice. At 0.5, 3, 6, and 24 h after the treatment, blood, heart, lungs, kidney, brain, liver, spleen, ovaries, and tumor were collected and weighed. To each organ, previously weighed, were added 3 mL of 65% HNO_3_. The organs were then kept for 3 h at room temperature in closed tubes and then for 24 h at 60°C. Afterwards, 2 mL H_2_O_2_ were added to the sample and heated to 60°C. Finally H_2_O was added up to a final volume of 10 mL and filtered with 0.22 µm filters. Blood serum was diluted 1∶20 and measured by using ICP-MS. Blood cells were resuspended in 6 mL of 65% HNO_3_ and 2 mL of H_2_O_2_ and heated in a microwave oven (Milestone Ethos 1) as follows: 2 minutes at 85°C, 3.5 minutes at 135°C, and 15 minutes at 230°C. After cooling down, H_2_O was added up to 10 mL final volume. The resulting solutions of the above procedures were directly measured using ICP-MS for the determination of Pt and Au (see Supplementary Information for instrument operating details).

### Kidney Histology

Proximal tubular degeneration was induced in mice by three consecutive ip injections (days 0, 3, 6) of 5 mg cisplatin kg^–1^ mice. The same dosage was applied in the case of AuNP-cisplatin, while control mice received no treatment. Animals were sacrificed three days after the last injection. Mouse kidneys and spleen were immersion-fixed in 10% neutral buffered formalin for 24 h and embedded in paraffin routinely. Sections (4 µm thick) were mounted on microslides and stained with hematoxylin-eosin. The specimens were examined and photographed using an Olympus PROVIS AX70 microscope (Olympus, Tokyo, Japan) equipped with an Olympus DP70 camera without prior knowledge of the applied experimental protocol.

### Therapeutic Efficacy in vivo

Mice with severe combined immunodeficiency (SCID) between 8 and 14 weeks-old were used to grow xenotransplant flank tumors, one per mouse, by subcutaneous injection of 20×10^6^ A549 tumor cells. For monitoring, tumors were measured three times weekly and the tumor volume was determined by the formula V = (A×B^2^)/2, where A is the largest diameter and B is the shortest diameter measured by caliper. After four to five weeks, once the tumor volumes were ≥100 mm^3^ and mean tumor size had reached 300 mm^3^, mice were divided into four groups of eight mice for treatments, to minimize weight and tumor-size differences. Tumor-bearing mice were treated by injection of 3 mg free cisplatin kg^–1^, 1.25 mg conjugated cisplatin kg^–1^, and controls were maintained without treatment. Treatments were administered in mice anesthetized by ip injection of 2,2,2-tribromoethanol-2-methyl-2-butanol (Avertine®, Sigma Aldrich) twice (day 0 and 3). Mice were monitored for a maximum of 10 days after first dose to avoid excessive tumor load, and mice weight and body weight loss were also monitored according to good laboratory practices to check excessive toxicity of treatments. Complete tumors of all animals were extracted and weighted at endpoint of efficacy studies.

Another round of mice was studied for a longer treatment period. Mice were injected subcutaneously with A549-luc-C8 cells (Caliper); 0.5×10^6^ cells). Tumors were grown over nine days before starting the treatment. Tumor growth was monitored every third day by using an *in vivo* imaging system (IVIS® Spectrum, Caliper), 150 mg D-Luciferine (kg mice)^−1^ was applied ip 5 minutes before scanning. Mice were placed under the CCD camera and kept under isoflurane anaesthesia (1.5–2% ) during the measures. Five mice received three ip injections of 1.5 mg cisplatin kg^–1^, five mice received the same amount of cisplatin conjugated to AuNPs and five mice, controls, were maintained without treatment.

## Results and Discussion

### AuNP Synthesis and Functionalization

We propose the use of cisplatin attached to 13 nm mercaptoundecanoic acid (MUA)-capped AuNPs via a pH-dependent coordination bond, as an efficacious antitumor drug. The bond between carboxylic acid and Pt is stable under physiologic conditions, but it is broken at acidic pH. It is well-known that NPs are internalized via an endocytic pathway [62]. Since the pH within the endosomes decreases, the release of cisplatin after internalization by cells is promoted. The active drug (aquated cisplatin) is able to diffuse out of the endosome and reach the nucleus. In fact, endosomal release has been previously postulated as an advanced mode of cellular delivery [63], [64]. Such processes, pH-sensitive release of drugs, have been proposed often in chemotherapy, for example, by encapsulation of a drug in a pH-sensitive polymer and subsequent release of the drug in the vicinity of the tumor, because of the lower pH found there [63]. In our case, the low pH at the tumor (6–7) does not cause liberation of the drug. A lower pH (≈5) is needed to break the coordination bond between MUA and cisplatin. Moreover, simultaneous monitoring of vehicle and drug biodistribution was possible due to the inorganic nature of both, and this can be correlated to the lack of systemic toxicity. Tracking both the NPs and the drug will help in the understanding how the nanocarriers are processed by the organism.

Conjugates were carefully prepared regarding size, drug loading, surface charge, and hydrophilicity, as well as stability even at high concentrations of AuNPs. Cisplatin doses in humans are around 1–3 mg of cisplatin per kg of body weight. For treatment in mice (about 30 g body weight) and taking into account that we can load around 500 cisplatin molecules per NP (which leads to the highest cisplatin coverage density to our knowledge in similar NPs), we need to inject about 750 µL of the conjugates solutions with concentration of NPs as high as 0.5 µM. The concentration is a serious issue since larger volumes cannot be injected into the animals, and we can hardly further increase conjugate concentration without compromising colloidal stability.

**Figure 2:**
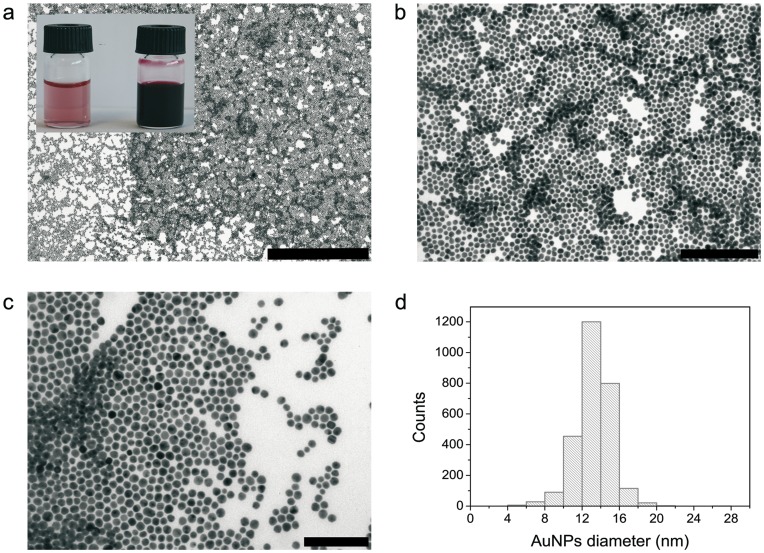
Transmission Electronic Microscopy (TEM) images of MUA-capped AuNPs. (a, b, c) Representative images of AuNPs. Scale bar represents 1 µm, 200 nm, and 100 nm respectively. (d) A narrow size distribution (13.3±1.9 nm) of AuNPs is observed. The appearance of the AuNPs as synthesized and after concentration process is shown in the inset in (a).

Biocompatible sodium citrate AuNPs were synthesized with a narrow size distribution (13.3±1.9 nm) and then modified with a MUA self-assembled monolayer (SAM) and concentrated by a destabilization–precipitation–resuspension process (Figure 2). Briefly, glycine buffer was added to decrease the pH to 2.6 to protonate MUA carboxylic acids. After removing the supernatant, MUA-capped AuNPs were resuspended in tricine buffer at pH 8 concentrating the NPs up to 2.75×10^14^ NP mL^–1^ to attain reasonable concentrations for the *in vivo* studies. The MUA SAM provided not only electrostatic stability to the system (ζ-Potential: –48.5 mV), but carboxylic groups for further linkage to [Pt(H_2_O)_2_(NH_3_)_2_]^2+^ (aquated cisplatin). It is known that following cisplatin ([PtCl_2_(NH_3_)_2_]) administration the chloride ligands are slowly replaced by water molecules, in a process termed aquation. The aqua ligands in the resulting [PtCl(H_2_O)(NH_3_)_2_]^+^ and [Pt(H_2_O)_2_(NH_3_)_2_]^2+^ are more labile than Cl^–^, activating the drug and allowing the platinum atom to bind to bases on DNA [39]. The [Pt(H_2_O)_2_(NH_3_)_2_]^2+^ solution is obtained by adding a solution of AgNO_3_ to commercial cisplatin [Pt(Cl)_2_(NH_3_)_2_] to promote the exchange of Cl^–^ for H_2_O ligands. After purification by recrystallization-washing steps, the solid [Pt(H_2_O)_2_(NH_3_)_2_] (NO3)_2_ was obtained and dissolved in water before use. It is important to work with the aquated form of cisplatin since H_2_O is a better leaving group than Cl^–^ and allows formation of coordination bonds between the Pt molecule and the deprotonated MUA carboxylic groups on the NP, otherwise, the commercial cisplatin molecule will not bind covalently [60]. In our configuration, the reactive part of the drug is protected, which leaves the inert NH_3_ moieties exposed to the exterior. Thus, the drug is protected against plasma deactivation. pH control is critical during the whole conjugation process: the p*K*_a_ value of a MUA SAM is reported to be between 6–8 [65], therefore the working pH must be above this value to ensure colloidal stability. However, at higher pH values, aqua ligands of the aquated cisplatin can undergo deprotonation to give hydroxo complexes that are less reactive (p*K*_a1_≈5.5, p*K*_a2_≈7.3) [66]. Since the hydroxylation reaction is slow at pH<9, working at pH 8.3 ensures both colloidal stability and formation of the conjugate. Otherwise the conjugate would lose electrostatic stability (at pH<p*K*_a_ of MUA) or cisplatin would be unable to form a coordination bond (at pH>9) and it would be electrostatically absorbed, and immediately released when dispersed in high-ionic-strength media such as biological fluids [60].

**Figure 3:**
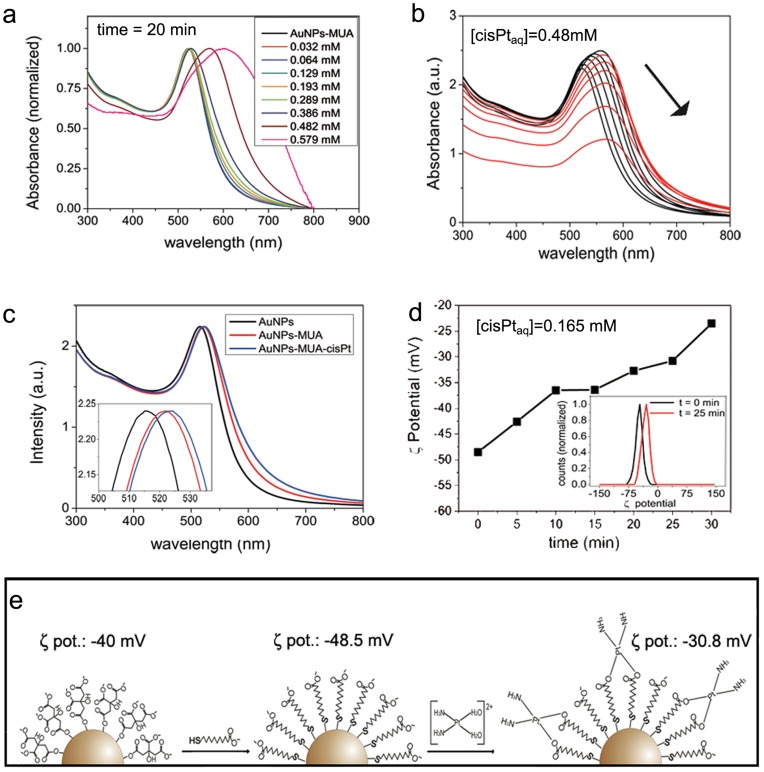
AuNP functionalization. (a) UV-Vis spectroscopy of conjugates with increasing aquated cisplatin concentrations shows that this concentration should not exceed 0.39 mM to guarantee colloidal stability. (b) When an excess of drug was introduced, the MUA charge became progressively quenched, which lead to aggregation of AuNPs, as denoted by the red-shift and further intensity decrease of SPR intensity in less than one hour. (c) Red-shift of the SPR peak at the working conditions: the initial peak of citrate-capped AuNPs shifted from 515 nm to 521.5 nm after MUA conjugation and to 523 nm after cisplatin conjugation. (d) Time evolution of ζ-potential after addition of [Pt (H_2_O)_2_(NH_3_)_2_]^2+^ indicates the quenching of negative charge on the MUA by the formation of a coordination bond between the carboxylic acid group and aquated cisplatin. Conjugates remained stable at ζ-potential values more negative than –25 mV. (e) Scheme showing the functionalization steps followed to obtain the cisplatin delivery system.

To achieve the maximum loading of cisplatin without compromising colloidal stability, aquated cisplatin was added in excess. The reaction was stopped by removing the excess of aquated cisplatin after 25 minutes by dialysis, when surface charge, as measured by ζ-potential, was −30.8 mV (Figure 3d). It is commonly accepted that values above +/−30 are needed to achieve colloidal stability [67]. This fact is in agreement with our observations that ζ-potential values that are less negative than –25 mV led to destabilization of the AuNPs and precipitation due to excessive quenching of the negatively charged carboxylic acid groups by cisplatin. A similar behavior was shown by Craig *et al.* when using a PEG linker which has a carboxylic terminal group as well [55]. Therefore, appropriate cisplatin concentration and incubation time are crucial to maintain colloidal stability (Figure 3 a, b, c). At the end, the loading of cisplatin on the NP as measured by inductively coupled plasma mass spectrometry (ICP-MS) was 42.3±0.8 mg Pt L^–1^, which represents an approximate loading of 470 cisplatin molecules per NP. Regarding conjugation, it has been recurrently observed that, in an excess of conjugating molecule without interfering species, homogeneous and dense monolayers of self-assembled organic molecules can form rapidly on the different NP surfaces [68]; therefore it is even more important to achieve homogeneous partial conjugation. It is likely that incubation of the AuNP-MUA with an excess of aquated cisplatin and then stopping the reaction by removing the excess of cisplatin by dialysis favors the formation of homogenously loaded conjugates.

**Figure 4:**
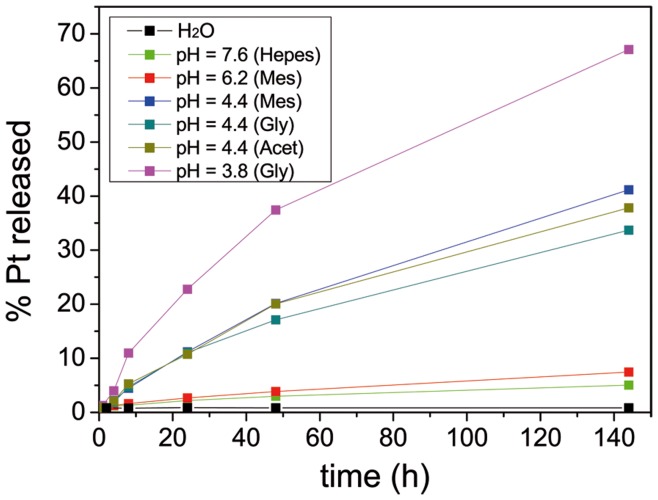
Cisplatin release vs. pH in physiological conditions. (high-ionic-strength media with 20% BSA; BSA = bovine serum albumin). At neutral pH the release was almost negligible but it increased at acidic pH values.

When preparing NP solutions for *in vivo* applications, the difference between NP conjugation solutions and physiological media is important; the latter has higher electrolytic concentration and strongly buffered pH value. In this context, colloidal stability in physiological media and no loss of drug during its journey through the body should both be guaranteed for success of the *in vivo* targeting [16]. Prior to *in vivo* experiments, colloidal stability was assayed in full human blood by dynamic light scattering (DLS) and UV-Vis spectroscopy. In DLS measurements, no peaks indicating the presence of aggregates in blood were observed. The 12-nm shift of the AuNPs peak is due to the formation of a protein corona [11]. Moreover, the UV-Vis spectra of AuNP–cisplatin in human blood indicated that AuNPs did not aggregate in this medium. The strength of the coordination bond makes the link between carrier and drug stable under physiological conditions. Since this bond is pH sensitive, increasing the [H^+^] leads to hydrolysis of the MUA–Pt bonds, releasing the drug in its active form. Only 5% of the Pt was released from the AuNPs at pH 7.6 after 144 h, while values as high as 40% of the Pt were reached at pH 4.4, and 67% at pH 3.8 (Figure 4). One could expect that the presence of different nucleophilic species, as the presence of protein, would alter the AuNP-MUA-CisPt ↔ AuNP-MUA + CisPt equilibrium. However, release seems to be independent of the buffering species, as demonstrated by observation of similar behaviors in three different pH 4.4 buffers.

### Pt Cell Internalization and DNA Accumulation

**Figure 5:**
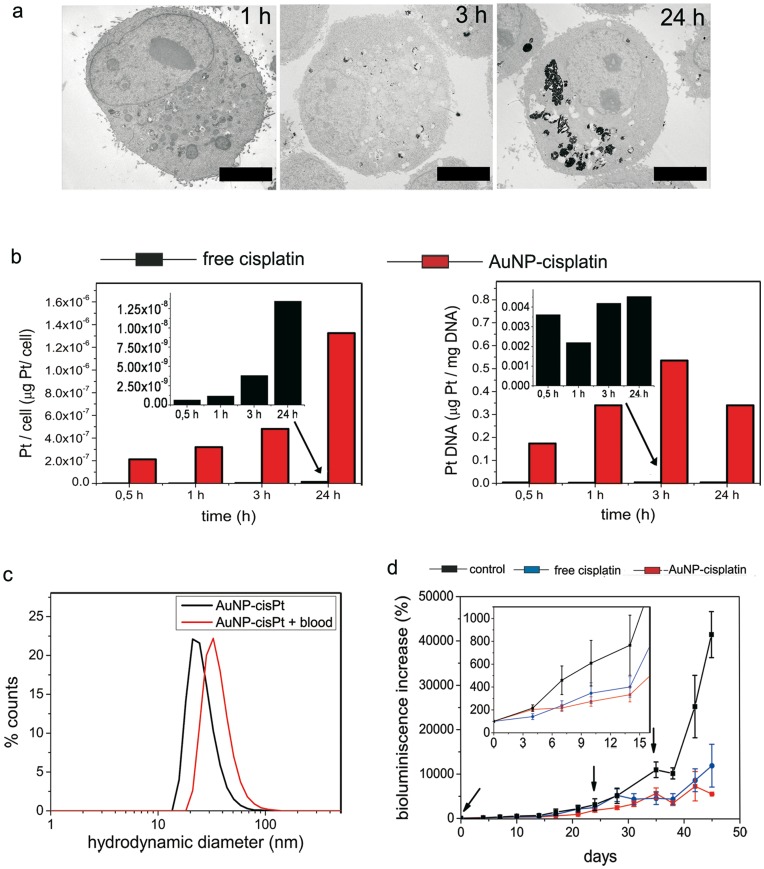
Cell internalization and accumulation, and therapeutic benefits *in vivo*. (a) Representative TEM images showing the internalization of particles at 1, 3, and 24 h. It is clearly shown that AuNPs were entrapped in vesicles that progressively fuse. The cell nucleus remained free of AuNPs. Scale bars represent 4 µm. (b, left) Cells show greater amounts of Pt when it enters attached to AuNPs than when in free cisplatin, especially at short time periods. (b, right) The acidic pH value in the endosomes promotes the release of cisplatin. Consequently the DNA targeting was also considerably improved by AuNP–cisplatin. (c) The colloidal stability is guaranteed in blood where the presence of aggregates is excluded by DLS. The 12 nm increase is due to the formation of a soft protein corona. (d) Increase in bioluminescence measured by IVIS which is proportional to the number of living cells in the tumor. 1.5 mg cisplatin (kg mouse)^–1^ injections were used in both treatments (day 0, 19, and 34). Arrows indicate injection days. Errors are standard error of the mean (n = 5).

Low cellular uptake of the drug may limit the efficacy of a chemotherapeutic treatment [69], therefore cisplatin cellular uptake and DNA binding were employed as signs of therapeutic activity. Adenocarcinomic human alveolar basal epithelial cells (A549) were treated with both free cisplatin and cisplatin conjugated to AuNPs (AuNP–cisplatin) at the same Pt concentration (1.7 µg mL^–1^). A faster uptake and higher cytoplasmatic levels of Pt were found in the latter case (Figure 5b). When cisplatin was conjugated to the AuNPs, cellular drug content was up to 300 times higher at short time periods than for free cisplatin, and levels up to 125 times higher were found in the DNA at 24 h (Figure 5b). As can be observed in transmission electron microscopy (TEM) images (Figure 5a), AuNPs mainly reside inside vesicles that evolve to form late endosomes and endolysosomes (vesicle size and number of particles per vesicle increase with time). Similar features were observed by confocal microscopy (Figure S2). These vesicles provide an acidic environment that promotes the release of cisplatin from the AuNPs, and its further escape from the endosomes to continue its journey towards the DNA. Note that the aquated form of cisplatin, due to its high lability, is expected not to travel far from the release point before reaching its target or being deactivated. Interestingly, the mechanism of drug entry into the cell is modified by conjugation: free cisplatin enters the cell mainly via passive diffusion through the membrane and by some transporter-mediated routes (e.g., copper transporters) [39], whereas cisplatin attached to AuNPs enters via an endocytic pathway. This active mechanism, together with the *cargo* effect, allowed the rapid accumulation of cisplatin. This rapid accumulation of cisplatin may enable us to overcome multidrug-resistance mechanisms that involve the overexpression of cisplatin efflux [70] proteins (e.g., P-glycoprotein) or facilitate DNA-repair mechanisms [40]. Additionally, the coating of the carrier with serum proteins, mainly albumin, may also favor conjugate uptake due to an overexpression of albumin receptors in tumoral cells [71]. Indeed, this is the claimed strategy for enhanced uptake of paclitaxel in Abraxane [72].

### Therapeutic Efficiency of AuNP–cisplatin

Animal models are particularly required when assaying the therapeutic efficiency of NPs as vehicles for drug delivery. Use of a nanocarrier will lead to an increased blood half-life and a higher accumulation in the site of action for the drug [73]. Thus, it is likely that a properly designed nanocarrier should have a better response *in vivo* than *in vitro,* with respect to the free drug in terms of concentration of active drug in the organ of interest. Additionally, issues such as tumor penetrability and toxicity might be modified as well and must be taken into account. One of the most common models consists of xenografted tumors implanted subcutaneously in Severely Compromised ImmunoDeficient (SCID) mice. If tumor progression has to be measured by size (e.g., using a caliper), initial tumors must be large enough to be measurable, in which case they are normally poorly irrigated and even may contain necrotic areas. Newer bioluminescent techniques (e.g. IVIS ®) are a good alternative to caliper measurements because they are more sensitive and therefore work properly with smaller tumours, but they depend on enzymatic activity. This technique has been used to follow tumorigenesis and response of tumors to treatment in animal models, since the expression of a bioluminescent marker specific of the implanted tumor cells is supposed to be proportional to the number of living cells [74]. However, environmental factors and therapeutic interferences may cause some discrepancies between tumor burden and bioluminescence intensity in relation to changes in proliferative activity; this must be taken into account along with the differing individual response to the tumor and the treatments.

In our experiments, 15 tumor-bearing mice were treated with 3 intraperitoneal (ip) injections of saline (control), free cisplatin, or AuNP–cisplatin. Doses of cisplatin were 1.5 mg cisplatin kg^–1^ in both treatments. Note that different concentrations are used throughout the work. The reason for these differences is the need to explore three different regimes: Tissue distribution, therapeutic window, and toxicity. In the first case, the doses are maximized to ensure detectable levels of gold and platinum in all tissues. For the therapeutic window, we explored the range of dosages which can prevent tumor growth effectively while staying in the safety range for the treatment (note that the treatment time is much longer than for biodistribution and toxicity). Otherwise, severe cisplatin-induced toxicity would have led to dead animals and consequently to the impossibility of comparing treatments. On the other hand, for the toxicity assays, we treated the animals with higher concentrations such as are generally used with cisplatin to induce toxicity in a short period of time. As can be observed in Figure 5d, the conjugation of cisplatin to the AuNPs does not affect its therapeutic effect since both the sample treated with free drug and that with drug conjugated to AuNPs showed no significant differences in tumor growth, as measured by bioluminescence, and both treatments showed decreased tumor growth with respect to the control. In a similar experiment, but starting the treatment when tumors were large enough to be measurable by caliper, the same tendencies were maintained (Figure S3). At the end of these treatments, tumors were extracted and weighed. The average weight of tumors in both treatments was 0.59±0.16 g for free cisplatin and 0.76±0.20 g when conjugated. These values are significantly lower than those of the control (1.81±0.54 g), with a Student’s t-distribution between the control and the treated mice with the conjugates of smaller than 0.05 (0.039).

Tumor penetration is known to be a limiting factor for cancer drug delivery. In fact, this has been recently identified as the major limitation in solid tumor treatment [75]–[77]. Despite this fact, conjugate sizes of around 15 nm have been reported to achieve good penetration and accumulation in the tumor [24], [44]. Obviously, cisplatin is smaller than the NPs and therefore its penetrability, in principle, could be higher. However the greater accumulation of active drug when attached to AuNPs (the drug is protected against deactivation by plasma proteins) and the possibility of successive treatments (if cisplatin toxicity is clearly reduced) may overcome the lower penetrability of the vehicles [75], allowing the progressive erosion of the tumor. Certainly, these dosing strategies could also be useful in the case of tumor resistance to cisplatin treatment. Finally, it is also worth noting that the metallic core of the AuNPs opens up the possibility of increasing tumor damage by using a combined therapy since they can efficiently act as photothermal therapeutic agents [2], [78] or as radiosensitizers [79], [80] which, together with surgery, are the common strategies used to treat cancer in the clinic.

### Biodistribution in Mice: Correlation of Biodistribution and Lack of Toxicity

Inside the body, the porous and diffuse frontiers of the different organs, which are all connected to the blood and lymphatic systems, have greatly differing transport abilities towards small molecules and nutrients, large proteins, or cells. Thus, the majority of drugs are normally transported through the small pores (6–8 nm) found in the continuous capillaries which are the most widely distributed throughout the organism [18], which causes unwanted side effects. For effective therapy, it is necessary to deliver therapeutic agents selectively to their target sites, avoiding non-target organs. Such selectivity is key for antitumoral drugs because of their extreme cytotoxicity. By vehiculating the drug using a nanocarrier, the distribution of the drug is controlled by the physicochemical properties of the nanoparticle [4], [17], [81] rather than those of the drug. Normally, *small molecule* drugs (the majority of drugs) have a short plasma half-life and are rapidly cleared by the renal system [18], which thereby greatly reduces the drug’s curative effect. Also, if escaping the kidney, NPs are easily endocytosed/phagocytosed, generally by circulating monocytes or fixed macrophages, which leads to their elimination from circulation and their simultaneous concentration in organs with high phagocytic activity. Although several factors such as core size and surface composition influence the fate of the NPs inside the organism, different types of NPs have been reported to be cleared within minutes from the bloodstream with a typical final biodistribution in the spleen, liver, and kidneys [20], [45], [67], [81]–[83]. Not only the NPs’ physicochemical properties (e.g., size, shape, and surface composition) determines the final fate of the NPs, but an important role is played by the array of serum proteins attracted by the NPs that form the well-known protein corona [11] which ultimately confers their biological properties [84]. In this regard, many studies indicate that particle size and surface chemistry (coating) govern translocation across epithelial and endothelial cell layers. In particular, regarding translocation of NPs, the studies summarized by Mehta et al. [85] and those performed by Heckel et al. [86] using intravenous administration of albumin-coated gold nanoparticles in rodents demonstrated receptor-mediated transcytosis (albumin-binding proteins). Similarly, polystyrene particles of 240 nm translocated across the alveolus-capillary barrier when coated with lecithin, whereas uncoated particles did not translocate [87].

**Figure 6:**
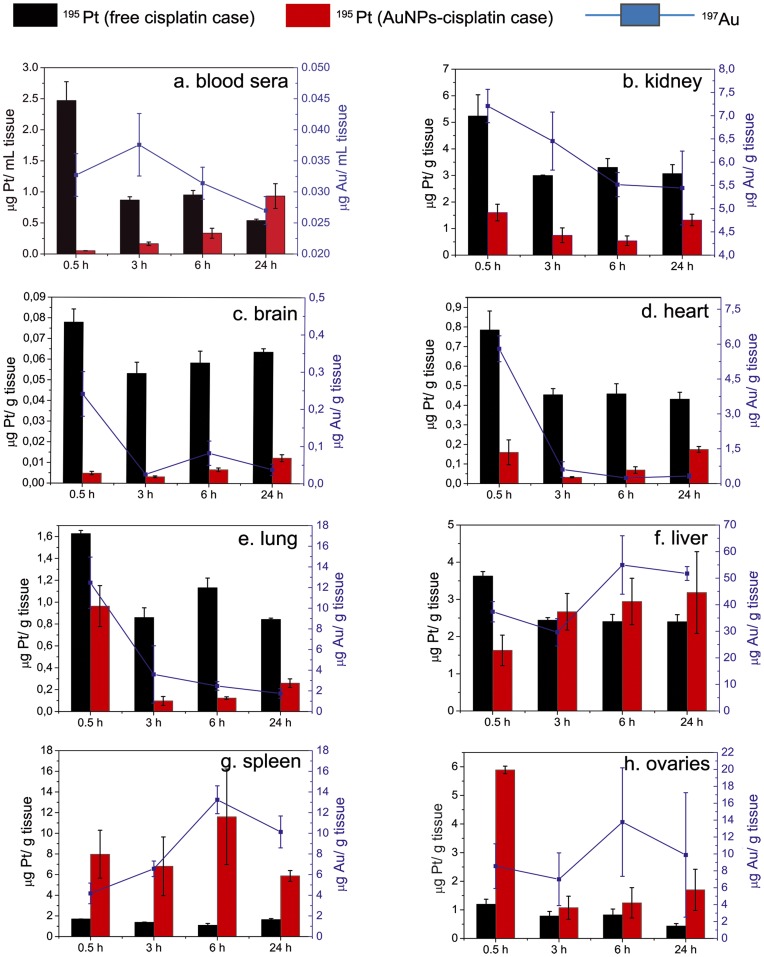
Biodistribution of Au and Pt arising from the treatment with free cisplatin (quantification of Pt, black) and AuNP–cisplatin (quantification of Pt and gold, black and blue respectively). The amounts of Pt and Au were analyzed in relevant organs at 0.5, 3, 6, and 24 h after injection by ICP-MS. Errors are standard error of the mean (n = 3). See text for extended analysis of this data.

**Figure 7:**
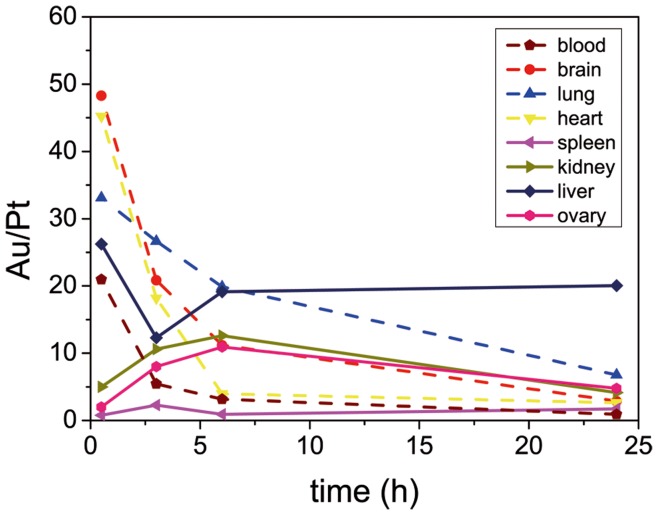
Time evolution of the ratio Au/Pt in different organs. Two different behaviors were observed: the one indicated by continuous lines (organs in which tissue macrophages are present) shows an increase of this ratio at the first times which indicates a depletion of Pt, likely due to the secondary release explained in the main text. Consequently, the other organs which are irrigated by continuous capillaries can take this Pt up and therefore the ratio decreases.

In our experiments, groups of 15 SCID human-tumor-bearing mice were used to assay the biodistribution of both the vehicle and the drug. The same amount of a high dose of free or AuNP-bound cisplatin was administrated to the mice (4 mg Pt (kg mice)^–1^) via intraperitoneal injection. This route facilitates the traffic of particles from the peritoneal cavity to the lymphatic system before the particles finally enter systemic circulation [88], from where they are distributed to the different organs. The amounts of both Pt and Au in blood, liver, spleen, heart, brain, lung, kidney, ovary, and tumor were measured using ICP-MS at 30 min, and 3, 6, and 24 hours after the injection (Figure 6). It is worth noting that from the crude readings it is impossible to determine if the observed Pt is still active, inactive bound to proteins, or attached to the NPs waiting to be activated. Free cisplatin is known to be removed from the circulation in two steps: an initial rapid renal clearance (less than 1 h) followed by a slow loss from the circulation of the cisplatin bound to plasma proteins (hours to days) [46], with less than about 3% reaching the tumoral cells’ DNA. Since the long-circulating cisplatin is mainly bound to protein and consequently deactivated [46], neither significant toxicity nor therapeutic benefits are expected from it [47]. This rapid clearance of free cisplatin, which is also observed from all organs in our work, differs considerably from what is observed in the case of the cisplatin bound to AuNPs. The amount of Pt in blood sera released from the AuNP-cisplatin conjugates (AuNPs were removed by centrifugation before measuring) was initially negligible (52.12±1.9 µg L^–1^). However, once the conjugates were processed, mainly by phagocytic organs (vide infra), there was a slow delayed release of Pt to blood that reached 932.25±343.4 µg L^–1^ at 24 h. In the rest of the organs it is common to see an initial decrease in Pt concentration, from 30 min to 3 hours and then an increase at 6 h and 24 h, likely of the non-conjugated form as the changes of the Au/Pt ratios in organs with time seem to indicate (Figure 7); this shows how the Au and cisplatin split and follow different pathways after being processed by cells. The higher the ratio, the less free cisplatin is present. Although the signal from conjugated and non-conjugated cisplatin is difficult to deconvolute, two different behaviors are clearly observed: organs where the ratio progressively decreases (those indicated by dashed lines) and organs where the ratio increases (those indicated by continuous lines). Note that the organs where this ratio increased are phagocytic organs where NPs accumulate. From these results we believe that some information regarding how nanocarriers are processed can be extracted: The stability of the link in blood is proved since the amount of free drug is initially negligible (in agreement with previous *in vitro* tests, Figures 4 and 5c). Then AuNPs-cisplatin start to accumulate preferentially in the phagocytic organs and are processed. From there the drug is released again to the systemic circulation in a process that we call secondary release, as the primary release is the release of cisplatin in the tumor cells. This secondary release can be considered an artifact of working with nanocarriers since it is known that, far away from the magic bullet concept, NPs are accumulated in phagocytic organs. However, there is a lack of cisplatin-induced toxicity due to this secondary release, which can be explained because cisplatin levels in blood are much lower than the achieved after free drug administration. Moreover the active form of cisplatin released from the NPs might be rapidly deactivated by plasma proteins, even faster than commercial cisplatin is deactivated, since it is more reactive. Consequently the drug coming from the secondary release is not expected to have significant therapeutic or toxic effects in our case, but this process should be taken into account when dealing with nanocarriers for drug delivery.

A detailed analysis of the biodistribution of the conjugates showed no evidence of accumulation of Au or Pt in the brain, heart, or lung other than that coming from the blood present in these tissues (Figure 6 c, d, e). 13 nm AuNPs are not expected to cross the blood–brain barrier [82], hence it is not surprising that the lowest Au content of all the analyzed organs was found in the brain. In all of these organs the amounts of Pt are 5-10-fold lower in the treatment with AuNPs-cisplatin than when treated with free cisplatin. A markedly different behavior was observed in liver and spleen (Figure 6 f, g) which are responsible for clearance of nanocarriers [20], [45], [67], [82], [83]. In fact, these –together with the ovary (Figure 6h) – are the only organs in which the Pt contents were higher in the case of AuNP–cisplatin treatments than for free cisplatin. These organs are highly phagocytic and will take up the AuNP–cisplatin conjugates and process them. Macrophages are found in the organs of the mononuclear phagocytic system (liver and spleen) as well as in the peritoneum (close to the ovary) and in the periphery of the kidneys. Accumulation of AuNP–CisPt conjugates in the ovaries, whose tumor is treated with cisplatin [38], is probably a consequence of the intraperitoneal administration. In fact, this route has been proposed for treatment of ovarian cancer with nanoparticles since they show a slower absorption profile into the lymphatic system than that of the free drug by this administration route [89]. It is also reasonable to think that in 24 h a significant amount of the conjugates is still in the peritoneum or lymph nodes, at the beginning of the journey that they perform during the treatments.

**Figure 8:**
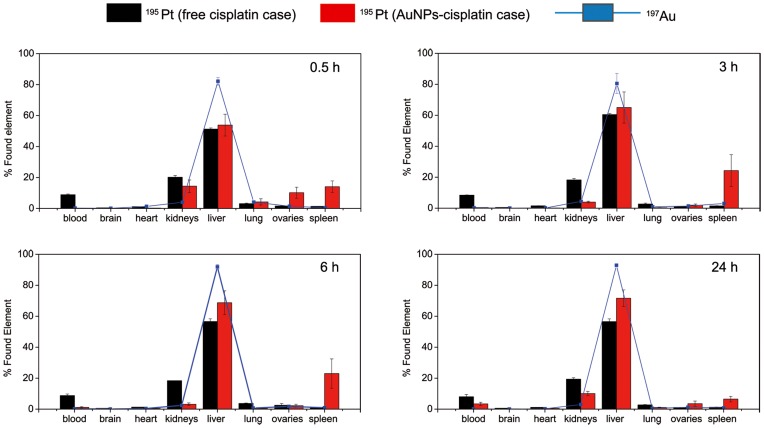
Biodistribution in function of the percentage of found elements for every organ at the four assayed times.

To observe the differential distribution in the measured organs, we also plot the relative amounts detected normalized to the total detected amounts at the different times (Figure 8). It is clearly observed that the cisplatin spared from the kidney seems to end up in the spleen, which deserves special attention in the case of vehiculated drugs.

**Figure 9:**
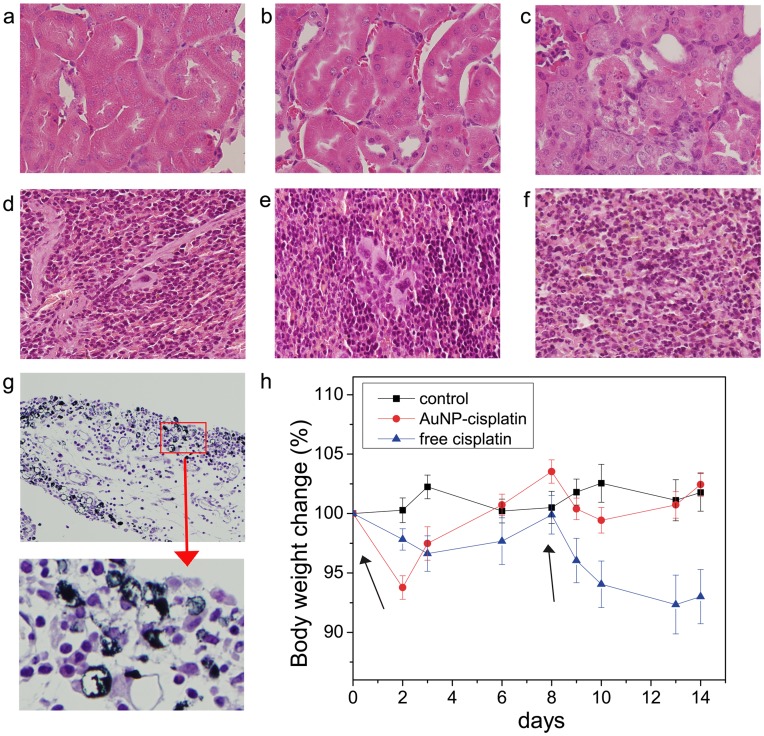
Toxicity of cisplatin. The appearance of histopathological changes in the proximal tubuli is evidence of nephrotoxicity. (a) Normal appearance of kidney section of control animals and (b) mice treated with AuNP-cisplatin. (c) Proximal tubular degeneration of animals taking high-dose cisplatin. Histological slides of spleen showed no signs of pathology in (d) control, (e) AuNPs-cisplatin, and (f) free cisplatin treatments. (g) AuNPs that reached the kidneys were trapped by peripheral macrophages. (Magnifications are x40 in a-f and x20 in d). (h) Body weight change of control mice and mice that received the same amount of free cisplatin or conjugated to AuNPs (4 mg kg^–1^, days 0 and 8). The arrows indicate the day of injection. Loss of weight and further recovery capacity is a clinical test to assay systemic toxicity. Errors are standard error of the mean (n = 5). Scale bars represent 100 µm in a, b, c and 200 µm in d.

**Figure 10:**
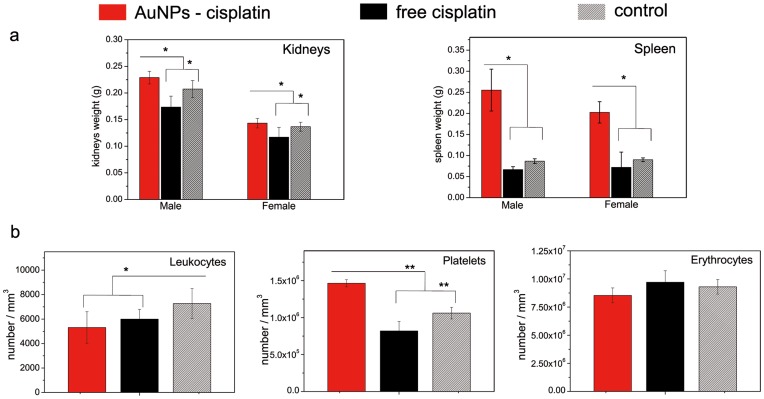
Kidney and spleen weights and hematology. (a) Weight of kidneys decreased only in the case of free cisplatin treatment, which agrees the previously observed cisplatin-induced toxicity. On the other hand, spleen weight increased in the case of AuNPs-cisplatin treatment, likely due to a temporal hyperplasia of the red pulp. (b) Leukocytes, platelets, and erythrocytes were also quantified and the organ showed no signs of anemia, but an increased number of platelets in the AuNPs-cisplatin, which is likely related to the hyperplasia of the spleen. * P<0.05, ** P<0.001.

The kidney deserves special consideration due to the high nephrotoxicity of cisplatin. Here, Pt levels were reduced considerably when AuNP–cisplatin was administered, compared to free cisplatin (Figure 6 b), at all times. Moreover, the AuNPs that reached the kidneys were trapped by peripheral macrophages, which thus diminished the potential damage induced by cisplatin (Figure 9g) as histopathology studies show (Figure 9 a, b, c). Histological analysis of the kidneys after treatment with free cisplatin and AuNP–cisplatin (21 days, 3 doses of 5 mg cisplatin (kg mice)^–1^ in both cases) revealed a lack of damage in the latter case, while in the case of free cisplatin, the kidneys were severely damaged. The morphological changes were consistent with cisplatin-induced acute nephrosis [90]. In addition to the observed absence of kidney damage in the mice treated with AuNP-CisPt, biochemical analysis did not show renal (blood urea nitrogen and creatinine levels) or liver (transaminases) damage (Table S1). What we observed was a modest lower leukocytes count for both treatments, slightly enhanced when the cisplatin is conjugated. Also there was no sign of anemia, and an increase of platelets in the case when the conjugates were employed (Figure 10 b).

When comparing the weight of the differently treated organs, clear reduction of the kidney weight was observed in the case of mice treated with free cisplatin; this is consistent with the observed histological damage. A significant increase of weight of the spleen treated with NPs (Figure 10 a) was also seen. To exclude any spleen toxicity induced by the nanoparticles, histological analysis of the spleen was also performed. Representative microscopy images of spleen slices of the control, the sample treated with free cisplatin, and a sample from the AuNP-cisplatin treatment do not show any adverse effect of the treatments in this organ; no damage or inflammation (Figure 9 d, e, f). Pathologists observed no significant differences in morphology between control, free cisplatin, and AuNP-cisplatin treated samples. In the case of AuNPs one could observe them accumulated in macrophages in the periphery of the spleen, as was previously observed in the kidneys. When using the conjugates, no morphological changes were observed, but a diffuse hyperplasia was observed, which manifested in the weight increase; the most significant alteration was the increased number of megacariocytes (platelet precursors), which can be related to the observed increase of circulating platelets. The diffuse hyperplasia of the red pulp was attributed to extramedullary haematopoiesis, likely after anemia due to the cisplatin treatment. Both anemia and hyperplasia are temporary and sequential and are expected to remedy themselves as cisplatin is being processed.

Lack of systemic toxicity in long-term treatment is normally assayed by measuring the body-weight loss from three groups of mice that received no treatment (control), free cisplatin, or AuNP–cisplatin (three injections of 5 mg cisplatin (kg mouse)^–1^ in both cases; in a 13 day experiment; Figure 9 h). After the initial weight loss in both treatments, weight recovery was most evident in mice treated with AuNP-cisplatin. More significantly, after the second dose, mice treated with free cisplatin decreased in weight more dramatically and showed no signs of recovery, which can be attributed to the cumulative toxic effects of cisplatin [91], whilst the mice treated with AuNP-cisplatin experienced a smaller weight decrease, a rapid recovery, and no evidence of any memory effects.

Although the lack of cisplatin-induced toxicity has been proven here when cisplatin is attached to the nanoparticles, a deeper understanding of nanoparticles’ inherent toxicity might be required before this technology can be used in clinical trials. There is controversy regarding the *in vivo* toxicity of NPs and the parameters that play a role in the NP-induced toxicity [92]. Some work found no toxic signals after AuNP administration [93] or small alterations in the biochemical markers due to metabolization of the AuNPs, or indicating a temporal inflammation [94]. On the other hand, it is believed that the dysfunction of major organs can be related to the presence of NPs at the site of abnormalities. For that reason, there are many studies regarding toxicity of AuNPs in spleen and liver, which are generally accepted to be the organs with the highest accumulation of AuNPs. Liver toxicity, when found, seems to be associated to an hyperplasia of Kupffer cells that induce an acute inflammation with neutrophil influx [82]. This acute inflammation is a transient response due to insult of AuNPs, however apoptosis and necrosis of hepatocytes, as well as accumulation of AuNPs, could be related to toxic effects [95]. Regarding the spleen, macrophages of the periphery seem to be involved in the uptake of NPs by this organ, thus leading to a temporal inflammation of the spleen. However, in other cases a loss of weight has been observed after intravenous administration [96]. White pulp aberration has been also observed [97]. No general trend can be extracted from the current literature, but it is clear that there are some parameters that have to be taken into account before using AuNPs as a vehicle for medical applications: i) size of the vehicle, since it will determine the clearance rate and the biodistribution [97]; ii) surface composition; it has been seen that by changing the surface composition of AuNPs the toxicological profile might be different [98]; iii) dose and administration route obviously will play a role in the potential toxicity. Here it is important to remark that the physicochemical properties that will influence the biological activity of the nanoparticle should be assayed in physiological conditions, since a change of these properties could happen in complex media such as biological fluids. Therefore for every AuNP-conjugate, a toxicology study must be done to prove its usability in medical applications.

### Conclusions

AuNP–cisplatin colloids were designed and prepared to be highly stable in physiological conditions to ferry the antineoplastic drug cisplatin towards its target, sparing the kidney. The conjugate has a size and a surface charge similar to those of proteins in serum to ensure its vehicular properties in physiological conditions, without interfering with cisplatin’s mechanism of action. This conjugation translates into a greater uptake and better DNA targeting of the drug, as proven by the *in vitro* experiments. *In vivo*, the vehiculization of the drug using AuNPs dramatically modified its biodistribution in mice, avoiding organs where cisplatin is known to be toxic while maintaining therapeutic benefits. Moreover, the drug made the journey through the body protected against deactivation by plasma proteins, and thanks to the pH-sensitive link, the active form of cisplatin was only released after cell internalization. It is also shown that any drug that did not reach the target was mainly processed by the liver and spleen, which allowed the delayed release of cisplatin. However this cisplatin is centered at the release point and rapidly deactivated (aquated cisplatin is more reactive than standard cisplatin). The pharmacological modifications –decrease in toxicity but not in therapeutic benefits– may also allow prolonged treatment.

In summary, the active mechanism of cisplatin, its pharmacodynamics, and binding to DNA and impedance of cell division, are not modified by its attachment to and vehiculation with a gold NP, while its pharmacokinetics and biodistribution are significantly modified.The major contribution of nanotechnology for delivering very effective drugs, such as cisplatin, is not to increase their therapeutic capacity, but better direct the drug to its target. Therefore, before the old dream of the magic bullet can be attained, a significant decrease of the drug’s toxicity, achieved here by modifying its pharmacokinetics properties, would represent a breakthrough in cancer treatment.

## Supporting Information

Figure S1 **Different cellular internalization of conjugate and free drug (not drawn to scale).** AuNPs-cisplatin are internalized via an endocytic pathway, hence cisplatin is only released at the acidic pH of the endosomes. Moreover AuNPs protect the drug from being deactivated by plasma proteins. Free cisplatin mainly enters via diffusion through the cell membrane. Inside the cytoplasm the interchange of Cl^−^ for H_2_O molecules takes place and the active drug is then formed.
(TIFF)

Figure S2 **Confocal microscopy of A549 treated with AuNP-cisplatin.** Cells were incubated with AuNP-cisplatin for (a) 30 min, (b) 1 h, (c) 3 h, and (d) 24 h. Afterwards, they were processed by staining nuclear DNA with DAPI, and α-tubulin microtubules with monoclonal mouse anti α-tubulin antibody and goat anti mouse antibody conjugated with Alexa 488. Nanoparticles appear as red dots because of their ability to scatter light [99]. A LEICA TCS SP2 AOBS Spectral Confocal system was used for image processing. There is evidence of internalization with time, and no nanoparticles were observed in the nucleus. Also, as time passes and nanoparticles are processed, deposits of NPs are observed. Some of those extracellular deposits may also occur due to the cell processing for microscopy.
(TIFF)

Figure S3 **Therapeutic efficiency and body weight change of short treatment.** (a) Differences in tumor volumes measured by caliper after two consecutive injections (day 0 and 3) of 3 and 1.5 mg cisplatin (kg mouse)^–1^ each of free cisplatin and AuNP–cisplatin, respectively. The antitumor activity of cisplatin was maintained after the drug was conjugated to the AuNPs. Tumor catch-up was also observed. (b) A large body-weight loss was caused by the high dose of free cisplatin (6 mg kg^−1^). This loss was not observed in the case of AuNPs-cisplatin. However the primary effect was not significantly different in both treatments.
(TIFF)

Table S1 **Analysis of relevant biochemical markers.** Aspartate transaminase (AST) and Alanine transaminase (ALT) levels indicate that there is no evidence of liver dysfunction. The renal function is usually determined by the levels of Blood Urea Nitrogen (BUN) and creatinine. Although the BUN seems to be higher than levels reported in other works [100], it should be noted that there is no difference between the control and treatment with AuNPs-cisplatin. Total protein and albumin are also indicators of hepatic function.
(PDF)

Text S1 **Experimental Details.**
(DOCX)
