# Supplementary figures and images for: Detoxifying Antitumoral Drugs via Nanoconjugation: The Case of Gold Nanoparticles and Cisplatin
Source: PLoS One. 2012 Oct 17;7(10):e47562. doi: 10.1371/journal.pone.0047562 (PMC3474726; doi:10.1371/journal.pone.0047562)

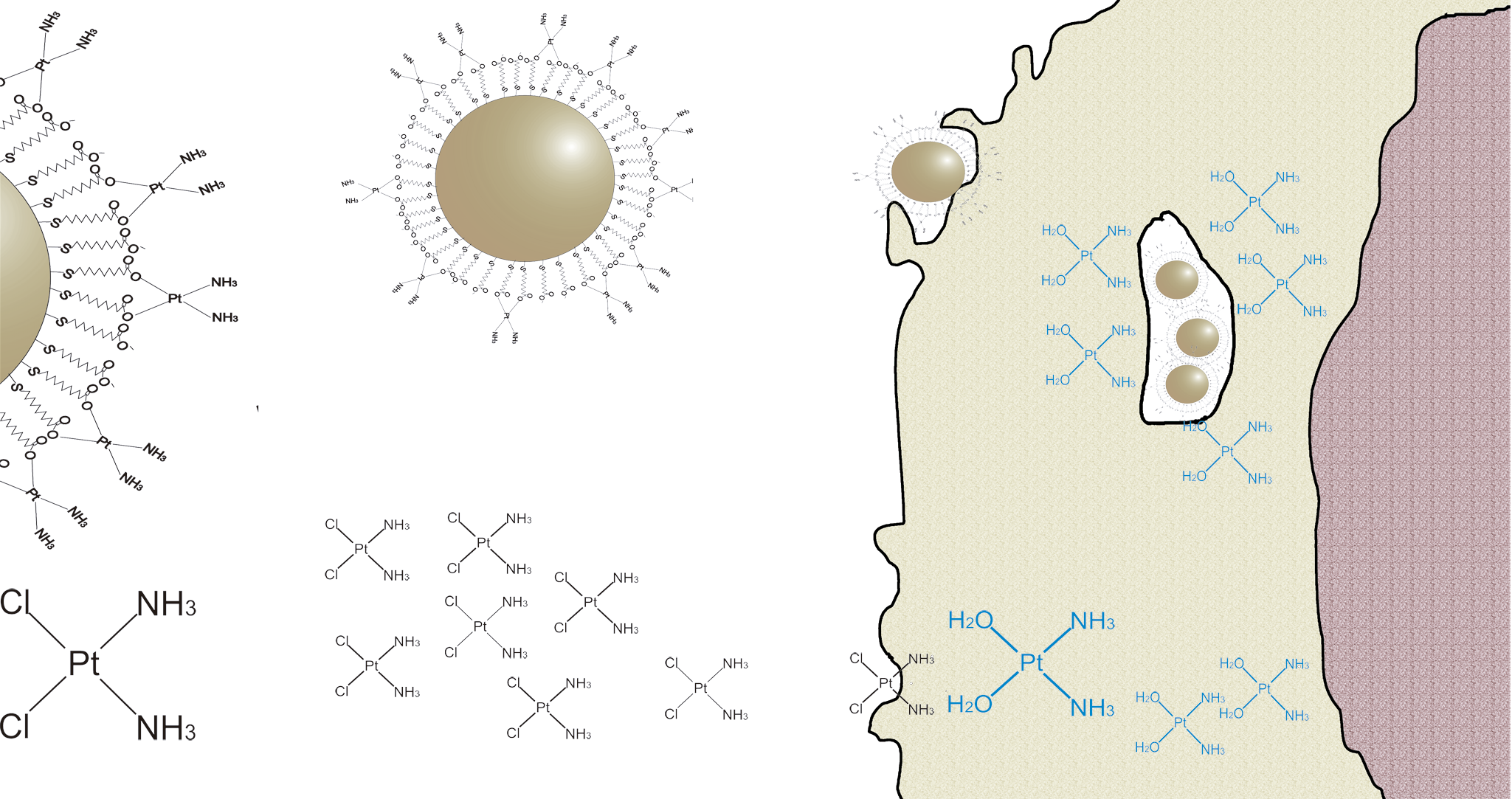

Supplement: Figure S1 — Different cellular internalization of conjugate and free drug (not drawn to scale). AuNPs-cisplatin are internalized via an endocytic pathway, hence cisplatin is only released at the acidic pH of the endosomes. Moreover AuNPs protect the drug from being deactivated by plasma proteins. Free cisplatin mainly enters via diffusion through the cell membrane. Inside the cytoplasm the interchange of Cl− for H2O molecules takes place and the active drug is then formed. (TIFF) [file pone.0047562.s001.tiff]

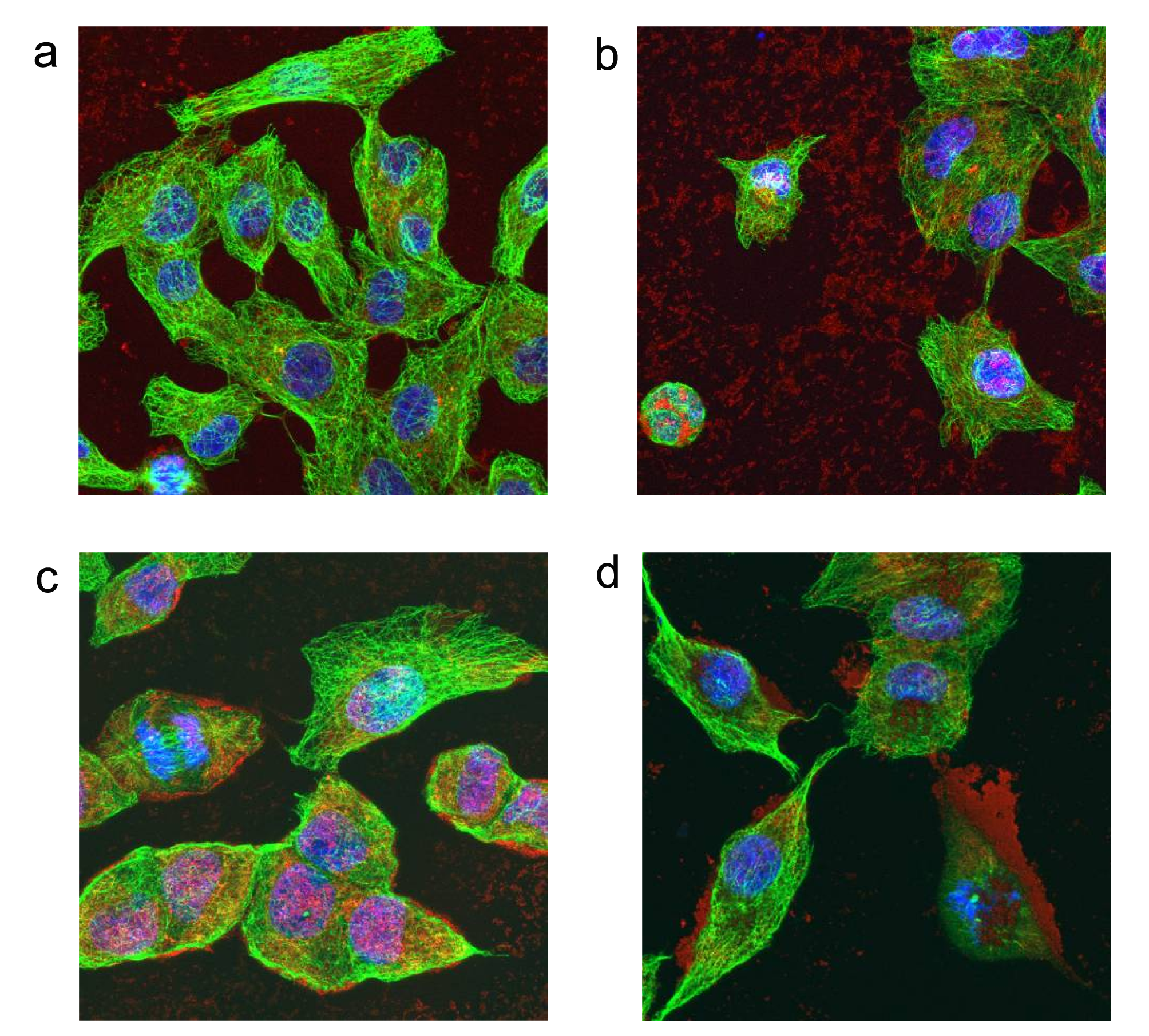

Supplement: Figure S2 — Confocal microscopy of A549 treated with AuNP-cisplatin. Cells were incubated with AuNP-cisplatin for (a) 30 min, (b) 1 h, (c) 3 h, and (d) 24 h. Afterwards, they were processed by staining nuclear DNA with DAPI, and α-tubulin microtubules with monoclonal mouse anti α-tubulin antibody and goat anti mouse antibody conjugated with Alexa 488. Nanoparticles appear as red dots because of their ability to scatter light [99]. A LEICA TCS SP2 AOBS Spectral Confocal system was used for image processing. There is evidence of internalization with time, and no nanoparticles were observed in the nucleus. Also, as time passes and nanoparticles are processed, deposits of NPs are observed. Some of those extracellular deposits may also occur due to the cell processing for microscopy. (TIFF) [file pone.0047562.s002.tiff]

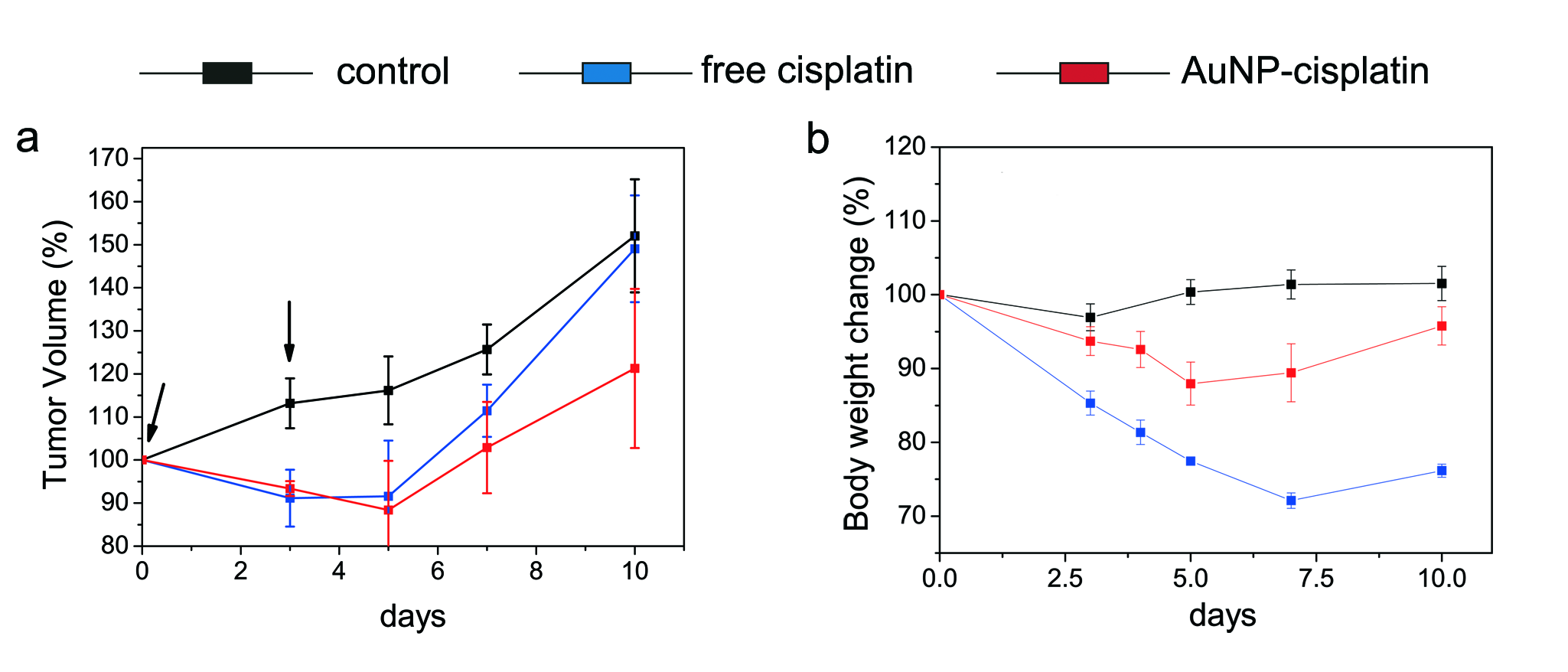

Supplement: Figure S3 — Therapeutic efficiency and body weight change of short treatment. (a) Differences in tumor volumes measured by caliper after two consecutive injections (day 0 and 3) of 3 and 1.5 mg cisplatin (kg mouse)–1 each of free cisplatin and AuNP–cisplatin, respectively. The antitumor activity of cisplatin was maintained after the drug was conjugated to the AuNPs. Tumor catch-up was also observed. (b) A large body-weight loss was caused by the high dose of free cisplatin (6 mg kg−1). This loss was not observed in the case of AuNPs-cisplatin. However the primary effect was not significantly different in both treatments. (TIFF) [file pone.0047562.s003.tiff]
