# Supplementary material for: Detoxifying Antitumoral Drugs via Nanoconjugation: The Case of Gold Nanoparticles and Cisplatin
Source: PLoS One. 2012 Oct 17;7(10):e47562. doi: 10.1371/journal.pone.0047562 (PMC3474726; doi:10.1371/journal.pone.0047562)
Supplement: Table S1 — Analysis of relevant biochemical markers. Aspartate transaminase (AST) and Alanine transaminase (ALT) levels indicate that there is no evidence of liver dysfunction. The renal function is usually determined by the levels of Blood Urea Nitrogen (BUN) and creatinine. Although the BUN seems to be higher than levels reported in other works [100], it should be noted that there is no difference between the control and treatment with AuNPs-cisplatin. Total protein and albumin are also indicators of hepatic function. (PDF) [file pone.0047562.s004.pdf]

	Control		AuNPs-cisplatin	
	Mean	sd	Mean	sd
AST (UI L^{-1})	15.88	4.79	15.46	6.73
ALT (UI L^{-1})	13.64	2.99	13.71	0.41
ALP (UI L^{-1})	109.87	64.68	189.5	99.7
BUN (mg dL^{-1})	55.93	8.3	63.26	11.78
Creatinine (mg dL^{-1})	<0.5		<0.5	
Total protein (g dL^{-1})	13.8	4.28	11.81	3.95
Albumin (g dL^{-1})	7.85	3.64	9.47	3.98
