# Supplementary material for: Detoxifying Antitumoral Drugs via Nanoconjugation: The Case of Gold Nanoparticles and Cisplatin
Source: PLoS One. 2012 Oct 17;7(10):e47562. doi: 10.1371/journal.pone.0047562 (PMC3474726; doi:10.1371/journal.pone.0047562)
Supplement: Text S1 — Experimental Details. (DOCX) [file pone.0047562.s005.docx]

**Text S1. Experimental details**

**Synthesis and characterization of MUA-capped AuNPs.** AuNPs (13 nm) were synthesized following a seeding growth mechanism based on the standard method of gold salt reduction by citrate[[1-2](#_ENREF_1)]. Specifically, Au seeds (7.5 nm, 5.5 x 10^12^ NP mL^–1^) were synthesized by adding an aqueous solution of HAuCl_4_ (1 mL, 25 mM) to a boiling sodium citrate solution (150 mL, 2.2 mM). When the reaction was complete, the temperature was decreased to 90 ºC and HAuCl_4_ (1 mL, 25 mM) was added to the previously synthesized AuNPs. This step was repeated twice more in order to get the final AuNPs (13.3 nm, 5.5 x 10^12^ NP mL^–1^). The conjugation of MUA was carried out by adding 2.2 mL of a 10 mM MUA basic solution to 50 mL of 13 nM AuNP solution and stirred gently overnight. The conjugates were concentrated up to 2.75 x 10^14^ NP mL^–1^ by a destabilization-resuspension step. 2.5 mL of glycine / HCl buffer (200 mM, pH=2.6) were added to 50 mL of MUA-capped AuNPs to destabilize them by protonation of the MUA SAM. The solution was then centrifuged (2 min, 2500 rcf) and the supernatant removed. The pellet was resuspended in 1 mL tricine buffer (50 mM, pH=8). The excess MUA was removed by a dialysis step (x1000, overnight). Surface plasmon resonance of AuNPs was determined at wavelengths from 300 - 800 nm. Particle size was measured by Transmission Electron Microscopy (TEM) imaging (Jeol 1010) and the hydrodynamic diameter by Dynamic Light Scattering (DLS) (Zetasizer nano ZS 90, Malvern Instruments).

**Synthesis of [Pt(H_2_O)_2_(NH_3_)_2_](NO_3_)_2_.** A solution of AgNO_3_ (169 mg, 1 mmol) in H_2_O (2.5 mL) was added dropwise to a suspension of cisplatin (150 mg, 0.5 mmol) in H_2_O (2.5 mL). A white solid (AgCl) precipitated and the yellow color of the initial mixture vanished after completing the addition. The resulting suspension was heated to 50 ºC for 1 h and AgCl was then removed by centrifugation. The supernatant solution was evaporated to dryness and the residue recrystallized from an ethanol/water mixture. Yield: 174 mg (89 %). ES-MS (MeOH): 263.79 [Pt(NH_3_)_2_(H_2_O)_2_ – H^+^]^+^, H_9_N_2_O_2_Pt^+^, calc. 264.03. IR (cm^–1^): 3283 (m), 1492 (m), 1384 (s), 1292 (m), 1263 (m).

**Conjugation of [Pt (H_2_O)_2_(NH_3_)_2_]^2+^ to MUA-capped AuNPs.** 5 μL of [Pt (H_2_O)_2_(NH_3_)_2_](NO_3_)_2_ aqueous solution (16.9 mg mL^–1^) were added to 1 mL of MUA-capped AuNPs (13 nm, 2,75 x 10^14^ NP mL^–1^) at pH 8.3. The solution was gently mixed for 25 minutes and the reaction was stopped by removing the excess of cisplatin derivative by dialysis (x1000, overnight). ζ potential measurements at different reaction times were performed using a Zetasizer nano ZS 90 (Malvern Instruments). Quantification of cisplatin loading was performed by using Inductively Coupled Plasma Mass Spectroscopy (ICPMS).

**Operating details for ICP-MS.** ICP-MS (BRUKER 820-MS) working conditions were:

Flow parameters (L min^-1^): plasma flow (17.0), auxiliary flow (1.65), sheath gas (0.19), nebulizer flow (1.00).

Torch alignment (mm): sampling depth (5).

Other: RF power (1.40 kW), pump rate, (5 rpm), stabilization delay (25 s).

Ion optics (volts): First extraction lens (-40), second extraction lens (-190), third extraction lens (-289), corner lens (-242), mirror lens left (42), mirror lens right (26), mirror lens bottom (47), entrance lens (-2), fringe bias (-2.5), entrance plate (-35), pole bias (0.0).

Scan mode: Peak hopping

Sampling time: 150 s

Dwell time: 30000 μs

Calibration standards were prepared in 5 % HNO_3_ at concentrations of 0.25, 0.5, 1, 2, 5, 10, 25, 50 and 100 µg L^-1^ of platinum (Fluka) and 10, 25, 50, 100, 250, 500 and 1000 µg L^-1^ of gold (Panreac). Iridium (20 µg L^-1^) was used as internal standard (Panreac)

REFERENCES

1. Bastús NG, Comenge J, Puntes V (2011) Kinetically Controlled Seeded Growth Synthesis of Citrate-Stabilized Gold Nanoparticles of up to 200 nm: Size Focusing versus Ostwald Ripening. Langmuir 27: 11098-11105.

2. Kimling J, Maier M, Okenve B, Kotaidis V, Ballot H, et al. (2006) Turkevich Method for Gold Nanoparticle Synthesis Revisited. J Phys Chem B 110: 15700-15707.
